# Opinion on the intersection of neurolinguistics, cognitive linguistics, and semantic rhetoric

**DOI:** 10.3389/fpsyg.2023.1101312

**Published:** 2023-01-19

**Authors:** Qing Zhang, Lei Liu

**Affiliations:** ^1^School of English Studies, Shanghai International Studies University, Shanghai, China; ^2^School of Foreign Studies, China University of Mining and Technology, Xuzhou, Jiangsu, China; ^3^Institute of Linguistics, Shanghai International Studies University, Shanghai, China

**Keywords:** semantic rhetoric, construal mechanism of discourse, cognitive neural mechanism, Annotation-Denotation Relevance-Inheritance Model, ERP studies

## Introduction

Recently, a synergy among neurolinguistics, cognitive linguistics, and semantic rhetoric has emerged due to the trend of interdisciplinary research. Liao and Meng (2022) co-authors a monograph to provide an essential supplement to the theoretical exploration of semantic rhetoric by means of the neurolinguistic approach, especially a breakthrough in the study of the connection between the source domain and target domain involved in the construction of semantic rhetorical discourse.

With postmodernism in mind, Liao and Meng (2022) constructs an effective analytical framework for studying Chinese semantic rhetoric, and selects samples of semantic rhetoric for observation, description, and analysis, and then reveals the construal mechanism of semantic rhetoric based on philosophy of mind and generative holism. Emphasis is placed on interdisciplinary research and the empirical methods of cognitive linguistics, pragmatics, and cognitive neurolinguistics are employed to explore the discourse mechanism of semantic rhetoric, which highlights valuable research fields for philosophy of language, human mind research, and language research related to artificial intelligence.

The research background lies in semantic rhetoric, which has a long history. Many researchers have explored rhetoric from the perspectives of classical rhetoric (Ricoeur, 1977), traditional rhetoric (Wang X. J., 2004), new rhetoric (Wang T. J., 2004), semantics and pragmatics (Chen, 2004), cognitive linguistics (Lakoff and Johnson, 1999; Sperber and Wilson, 2001), neurolinguistics (Wang, 2009), and so on. They have neatly clarified the complex situation of rhetoric, and accumulated a lot of achievements in the classification, description and normative application of rhetoric. However, research into the construal mechanism of semantic rhetoric requires further study. Cognitive neurolinguistics is the subject of a large body of research in semantic rhetoric, but these studies focus primarily on a certain kind of semantic rhetoric by means of ERP experiments, such as metaphor (Coulson and Van Petten, 2007; Lai et al., 2009; Weiland et al., 2014; Wang, 2019), pun (Coulson and Severens, 2007; Wang and Zheng, 2019; Liao et al., 2021; Wang et al., 2021), and so on. Zhang (2016) also uses ERP technology to explore the temporal processing and neural mechanism of representing and processing different kinds of idioms, thus enriching people's understanding of idiom processing in the brain. However, there is a paucity of research directly related to the cognitive neural mechanism of rhetorical semantic discourse. Only in the last few years has similar research emerged, covering a small scope, and few researchers have attempted to place semantic rhetoric in a unified linguistic theoretical framework and verify the framework using ERP experiments. In this way, the current study hopes to make a novel attempt to investigate the cognitive neural mechanism of rhetorical semantic interpretation.

The research motivation is to first introduce the analytical framework of the research on Chinese semantic rhetoric proposed by Liao and Meng (2022), reveal the mechanism of semantic rhetoric, and adopt this framework to analyze semantic rhetoric examples and indicate the feasibility of this framework. Second, the technique of ERPs from cognitive neuropsychology is adopted for experimental investigations to demonstrate the psychological reality of the cognitive neural mechanism of semantic rhetoric. Since there are many kinds of rhetoric in Chinese, and such a study cannot cover all kinds of rhetoric, this research focuses on semantic rhetoric. In conclusion, we believe that the manuscript's tentative investigations into the cognitive neural mechanism of construal semantic rhetoric from the perspectives of both theoretical construction and empirical psychological assessment have attracted academic attention, and are worth recommending to researchers in the areas of general linguistics, cognitive neuro-philology, psychology, rhetoric, and language education.

First of all, some basic concepts such as rhetoric and semantic rhetoric need to be clarified. According to Liao and Meng (2022, p. 4), rhetoric is a choice based on synonymous and/or formal expressions, semantic or pragmatic variation/deviation in order to arrive at the best verbal expression for communication in a given context. In other words, rhetorical discourse, like ordinary speech, is the selection of expressions made by the speaker based on the need to realize the communicative intention; the essential properties and underlying mechanism of the discourse as a whole are common to both ordinary discourse and rhetorical discourse. Accordingly, this study defines rhetoric as a conscious and intentional process of refining the verbal communicative discourse within a certain context. As a result, rhetoric sustains multiple attributes. They are verbal, purposive, cognitive, contextual and social, and have their own patterns. And the semantic rhetoric discussed in this manuscript refers primarily to rhetorical expressions constructed using “semantic variation/deviation” of concepts.

In Liao and Meng's (2022) introductory part, the difference between rhetoric in the broad and narrow senses is discussed, briefly combs the development of rhetorical research, and points out that it is most representative of the study of contemporary rhetoric from the point of view of cognition, which is rooted in the fact that language is metaphorical (Lakoff and Johnson, 1999). Liao and Meng (2022) reclassifies the eight types of semantic rhetoric—metaphor, metonymy, pun, irony, hyperbole, analogy, euphemism, and transferred epithet—into three categories: proximity-based (metaphor, analogy and transferred epithet), similarity-based (metonymy, irony and hyperbole), and both proximity-based and similarity-based (pun and euphemism) (He, 2013). The goal is to theoretically and empirically explore the cognitive neural mechanism of rhetorical semantic utterances, which is also clarified in this section. The study primarily adopts the information processing method in the brain, and uses experimental brain imaging results to certify theory, and investigates the specific methodology. First, it employs qualitative research methods, in particular abductive reasoning (Hopper and Traugott, 1993). It selects typical rhetorical expressions for observation and description, and attempts to give research hypotheses. The research constructs an analytical framework of relevant construal mechanisms to explain online construal, and the validity and appropriateness of the explanations are then used to highlight the applicability of the assumptions (Liao, 2011, p. 7). Second, it explores the cognitive neural mechanism of construal semantic rhetoric discourse through the use of ERPs as a high temporal resolution electrophysiological technology.

Liao and Meng (2022) focuses on the review of rhetorical research. Rhetoric is a discourse expression that is selected on the basis of synonymous expression and/or semantic variation in order to achieve the best in the current context. That is, rhetorical statements, like general statements, are expressive choices made by the speaker based on the need to realize communicative intentions. The essential features and underlying mechanisms of discourse, general discourse, and rhetorical discourse are thus interrelated. The contemporary research of rhetoric is interdisciplinary due to its intimate relationship with linguistics, cognitive science, philosophy, psychology, sociology, and anthropology. Fundamental questions about rhetoric make critical practice possible across the field of cognition. The purpose of their study is to investigate the cognitive neural mechanism of rhetoric, attempting to answer the following two meaningful questions. What is the construal machinery of semantic rhetoric? Can the construal mechanism of semantic rhetoric be tested psychologically?

Thereupon, Liao and Meng (2022) builds the framework for analyzing the construal mechanism of semantic rhetoric. The study begins with a detailed discussion of its theoretical underpinnings, including postmodern philosophical approaches in language studies, philosophy of mind, generative holism, cognitive science, cognitive pragmatics, cognitive neurolinguistics, and the like. The second is a discussion and revision of Liao's (2011, p. 88) construal framework of discourse under the Holistic Cognitive-Pragmatic Model (HCPM). It is currently updating and optimizing the Annotation-Denotation Relevance-Inheritance Model (ADRIM) to account for the construal of semantic rhetoric. ADRIM's theoretical framework provides a unified explanation for the construal mechanism of semantic rhetoric. Note that in the process of extracting features possible under mind-attachment, conventional relationships and context are essential. It is possible that ADRIM produces a unified interpretation in the construal mechanism of rhetoric at both the lexical and semantic levels, with a focus on research related to mind-related issues such as the “abstraction of possible functionality” and “gestalt transfer.” ADRIM is a semantic rhetoric discourse construal framework constructed by mending and optimizing the discourse construal framework based on the theory of philosophy of mind, generative holism and the Impartment and Inheritance of Connotation and Denotation. A brief description of the framework can be given as follows: The construing process of rhetorical semantic discourse is situated in a specific context and is dominated by intentionality. In the framework, it relies on proximity and/or similarity, and under the mental attachment restriction it infers the implied expression from the abductive cause of the explicit expression and then generates the communicative intent. Specifically, by virtue of proximity and/or similarity, Relevance-Inheritance reasoning can be used to construct the annotative and denotative relationship of the two things/events (A and B) that are involved in semantic rhetoric. Accordingly, a representation “A is B” is set up by A's endowment of (a) possible feature(s) that is availed by the mental-physical supervenience and that A does not normally have. Finally, referring to the holistic context, one can obtain a relatively complete expression and infer communicative intent (p. 83–84). This model is mainly used in the construe process of semantic rhetoric (such as metaphor, metonymy, pun, irony, hyperbole, analogy, euphemism, and transferred epithet discussed in this monograph), which usually involves the relationship between two domains of concepts.

Liao and Meng (2022) presents the preliminary ADRIM application process and performs the semantic rhetorical data analysis. It attempts to test the feasibility and operability of the ADRIM framework in a way by analyzing some instances of Chinese semantic rhetorical utterances. The eight types of Chinese semantic rhetoric reflect different proximity/similarity relations, but they all concentrate on semantic deviation from different perspectives. Each of these semantic variations may construct different relationships between things based on certain possible characteristics. Thus, it can be proven that the ADRIM framework constructed in this study can be effectively applied to the discourse mechanism analysis of semantic rhetoric, and possesses a strong rationality and operability property. In this process, extracting the possible features under the effect of mental-physical supervenience is the key step, which is also the focus of semantic rhetoric discourse construction.

The research is the demonstration of the technology of cognitive neuroscience experiments and the specific experimental design. The necessity and practicality of ERP experiments are clarified, and the choice of experimental research design for this study is discussed. First, the development of research in cognitive neurolinguistics is briefly introduced. In order to explain the rationality and operability of this experimental research as well as to explore the overall scheme of experimental ERP research. Currently, cognitive neuropsychological and linguistic techniques that are widely used in the field of language research are primarily eye tracking, fMRI, and ERPs. By introducing and comparing the three technologies, ERPs are found to have high temporal resolution, people can study the process of natural-state language comprehension, increasing the sensitivity of the task, as well as providing more abundant and efficient experimental data. Thus, ERP technology may provide a reasonable and efficient tool for studying the cognitive neural mechanism of rhetoric. The main components of syntactic processing are the Left Anterior Negativities (LAN) and the P600. The most prominent and significant features are the N400 and P600. N400 is the most classic and stable ERP component recognized by the academic community. It can not only reflect the semantic processing process in language understanding, but also indicate the construction process of context constraints on meaning. The greater the difficulty of the semantic processing and integration process, the greater the magnitude of the N400. P600 is the Late Positive Components (LPC) distributed across the central parietal area, which may reveal syntactic violation and reflect the process of syntactic integration. P600 may also reflect the situation of no syntactic errors but of difficult processing; In the case of true syntactic violations, the LAN appears before the P600, so the LAN shows the detection of the initial syntactic violation, whereas the P600 belongs to the subsequent syntactic error repairs (Wang, 2009, p. 156–159). Liao and Meng (2022) employs the embodied cognition model of language comprehension, namely index hypothesis theory, as a guiding framework, and refer to the dynamic acceptability/availability extraction research paradigm of language comprehension in order to explore the psychological reality of possible feature extraction in the process of rhetorical construal. At the same time, according to the particularity of the construal of semantic rhetoric and the experimental design principle of this experimental paradigm, the N400 components related to early semantic processing and the slow potential late positive (LPC) and other ERP components related to late semantic integration processing are the focal point of the current study.

Liao and Meng (2022) is devoted to the experiments. In these experiments, the ERP technique is used to verify the psychological reality of possible feature extraction. ESL students of Chinese-native speakers are recruited as experimental subjects. Metaphor, irony, and pun samples, which represent three kinds of relationships respectively—similarity, proximity, and both similarity and proximity (p. 158)—are selected as materials for the experiments. Here are the experiments and discussion: First, most of the results of the behavioral experiments are consistent with the psychological reality of feature extraction from possibility in the construal process of semantic rhetoric, and feature extraction opportunity plays a positive role in promoting discourse construal in semantic rhetoric; ERP components such as N400 and LPC, which show elements of semantic integration, lend support to the psychological reality hypothesis in the process of rhetorical construal of all levels. Second, the influence of opportunity feature extraction on the interpretation of both semantic and non-semantic rhetorical speech includes individual differences. Third, “possible feature extraction in semantic rhetorical comprehension” (p. 158) has a significant impact on the interpretation and processing of semantic rhetoric. Discussion of the three types of experimental cases above supports the following conclusions: First, possibility feature extraction highlights the process of mental processing and construction of rhetorical semantic understanding; Second, the accessibility of rhetorical discourse exhibits the gestalt effect. Third, extension, relevance, and inheritance of connotations are the central building blocks and important methods of rhetorical construal. In conclusion, it is worth paying attention to the fact that the influential factors in the processing of rhetorical semantic discourse are multidimensional, such that many factors, such as familiarity, salience, and context, do not affect its processing and construction alone, and these often interactively affect the processing of rhetorical expressions.

The key findings of this study can be summarized as follows. First, from the point of view of constructive postmodern philosophy, the “experience-oriented view” and the “sense-conscious view” of linguistic research, both lay the theoretical groundwork for studying the cognitive neural mechanism of rhetoric. Second, the gestalt effect carries over to rhetorical discourse comprehension, and the entire gestalt transformation is necessary in the process of construal of rhetorical discourse. Third, sentience is the consciousness ground of rhetoric, and the extraction of possible features is produced by the mental-physical supervenience of things. The representation of the linguistic world is the mental representation, the subjective mental image produced by the mental world in the face of the external world, and the unity of the subject and the object (Xu, 2011). Fourth, left and right brains are both activated in the rhetorical construction process, but exhibit asymmetrical features and play different roles. Fifth, there are many influential factors in the processing of rhetorical discourse, which have interactive effects on the processing process. Most importantly, Liao and Meng (2022) develops an integrated speculative and empirical research approach, and may provide a novel methodological alternative for rhetorical semantic studies in different languages.

Liao and Meng (2022) probes into the theories of applied cognitive linguistics and cognitive pragmatics comprehensively. On the one hand, it constructs the analytical framework of discourse construe mechanism of semantic rhetoric to carry out theoretical exploration. On the other hand, sample analysis and cognitive neuroscience experimental methods are used to verify the conclusions of the speculative research. This study provides important illumination and practical guidance for language teaching, especially the teaching of rhetorical discourse. First, the relevant enlightenment of semantic rhetoric and cognitive neural mechanism can provide important theoretical support for the teaching and research of Chinese as a mother tongue, so as to improve the expression ability of native Chinese speakers. Second, the conclusion of the study provides a theoretical and practical basis for the compilation of teaching materials, curriculum design and examination propositions. Third, it can supplement relevant theoretical principles and practical experience for the basic research and practice of Chinese-English/English-Chinese translation, especially the translation of the eight common semantic rhetoric utterances in English and Chinese mentioned above. Fourthly, it may provide relevant theoretical support and practical guidance for the study of children's semantic rhetoric discourse acquisition.

This research is still worthy of discussion and improvement. Given the limitations of the experimental conditions, most of the corpora involved in the study are solely Chinese rhetoric, therefore, more comparative multilingual research needs to be supplemented. All in all, this monograph explicates significant research orientations in the future for philosophy of language, language and cultural research, and human mind studies related to big data and artificial intelligence.

## Author contributions

QZ wrote the manuscript and LL revised it. All authors contributed to the article and approved the submitted version.
